# Step by Step Construction of Multifunctional Hollow Double Shell MNPs@MOF as a Powerful Tandem/Cascade Catalyst

**DOI:** 10.3389/fchem.2021.738736

**Published:** 2021-09-15

**Authors:** Shunli Shi, Ying Yu, Bingzhen Zhang, Yicheng Zhong, Lei Wang, Shuhua Wang, Shunmin Ding, Chao Chen

**Affiliations:** Key Laboratory of Jiangxi Province for Environment and Energy Catalysis, College of Chemistry, Nanchang University, Nanchang, China

**Keywords:** tandem/cascade catalysts, MNPs@MOF, hollow structure, accelerated mass transfer, imines

## Abstract

The development of efficient heterogeneous catalysts for one-pot tandem/cascade synthesis of imines remains meaningful and challenging. Herein, we constructed an Au/MOF catalyst featured hollow and double MOF shell nanostructure. Owing to its structural merits and acid-basic nature, the as-synthesized Void|(Au)ZIF-8|ZIF-8 catalyst exhibited an enhanced synergistically catalytic performance for tandem catalytic synthesis of imines from benzyl alcohol and aniline under air atmosphere and solvent-free condition. Its 170.16 h^−1^ of turnover frequency (TOF) was 2.5 times higher than that of the reported catalyst with the highest TOF value.

## Introduction

Imines (Schiff bases) are widely presented in natural products, bioactive compounds and pharmaceutical structures (Kobayashi et al., 2011; Nielsen et al., 2011). It is prevalent in the field of fine chemical, pharmaceutical, and chemical industries and plays an important role in the synthesis of N-containing heterocyclic (Dhakshinamoorthy and Garcia 2014; Bhaskaruni et al., 2020; Djakovitch et al., 2011) compounds as a nitrogen source (Shang et al., 2019; Chen et al., 2021; Long et al., 2017; Hong and Ye 2020; Chen et al., 2015). In particular, the implementation of one-pot tandem/cascade catalytic reactions to realize imines and their derivatives are of the greatest interest to chemical researchers (Huang et al., 2017; Wu et al., 2019; Wang et al., 2018). A number of Au NPs/carrier heterogeneous nano-catalysts such as Au/ZnAl_2_O_3_ (Wu, Sun*,* et al., 2019), Au/HAP (Sun et al., 2009) and Au/TiO_2_ (Kegnæs et al., 2010) etc. have been considerably studied for the construction of imines from oxidative self-coupling amines or selective coupling amine and alcohol under oxygen or even air atmosphere condition.

Generally, the key to improve the TOF value for heterogeneous tandem catalyzed synthesis of imine reactions lies in several factors. I) Highly dispersed fine Au nanoparticles (NPs) or Au-Pd alloys NPs can catalyze the oxidation of benzyl alcohol to benzaldehyde smoothly. In the presence of oxygen, the Au-H that formed from hydrogen extracted by the highly dispersed fine Au species is rapidly oxidized, leading to the catalytic cycle progress (Tsukuda et al., 2011; Parmeggiani and Cardona 2012). Alternatively, Au-Pd alloys NPS can also be constructed to realize the activation of oxygen for the purpose of enhancing hydrogen consumption (Yu et al., 2015; Soulé et al., 2013; Zhang et al., 2019). II) Alkaline auxiliaries (usually *t*-BuOK, NaOH and K_2_CO_3_ etc.) with the de-protonation effect can effectively assist Au NPs to extract hydrogen, thus greatly speeding up the process of oxidation reaction (Hoover and Stahl 2011; Li et al., 2007; Prati and Rossi 1998; Biella et al., 2002). III) The carrier materials with acidic active sites are conducive to the condensation reaction of benzaldehyde and aniline, thus achieving a one-pot tandem/cascade reaction (Zhang et al., 2020; Sun et al., 2009; Kegnæs et al., 2010). Strikingly, metal-organic frameworks (MOFs) possess tunable open metal centers, high surface area and acid/base active sites (Zhang et al., 2020; Huang et al., 2017), which endow them as the promising carrier candidate for tandem catalyzed synthesis of imine. As a matter of fact, several composite Au/MOFs catalysts (Chen et al., 2017; Wang et al., 2018; Gumus et al., 2021) have been developed that allow alcohol to be effectively coupled with amine in one-pot tandem/cascade catalysis reactions. Results demonstrate that the high dispersion of Au NPs and the active acid sites of MOFs are the key to the oxidative coupling of amine and alcohol. Further, the high surface area and pore volume of the carrier material is supportive of the adsorption of oxygen, increasing the concentration of oxygen in the reaction system (Gumus et al., 2021). As summarized in Supplementary Table S1, however, the low turnover frequencies (TOFs <80 h^−1^) and the economical inefficiency caused by the introduction of organic solvent or alkaline auxiliaries into the catalytic system limit the current catalysts in further industrial application.

**FIGURE 1:**
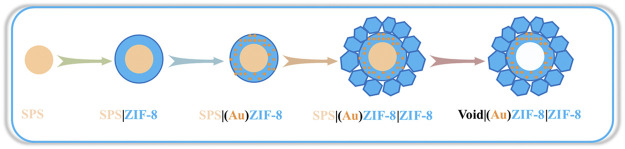
Growth procedure for the Void|(Au)ZIF-8|ZIF-8.

In view of this, we predict that the structure and property of Au/MOFs can be further optimized to improve the TOF values, and even to make the reaction conditions more moderate and economical. As well known, the hollow structure has been demonstrated to enhance the catalytic performance of catalysts due to the unique role in accelerating mass transfer at the nano-scale (Zhong et al., 2019; Wan et al., 2017). In addition, some MOFs possess basic active sites (Zhang et al., 2020), which can avoid using alkaline auxiliaries to make the reaction conditions more mild and economical. Therefore, we herein report an Au/MOF catalyst with hollow nanostructure featured double MOFs shells supported Au NPs (Void|(Au)ZIF-8|ZIF-8, Figure 1) as high selectivity heterogeneous catalyst for the one-pot tandem/cascade synthesis of imines. Typically, step by step construction of ZIF-8 shell tends to have both acid and base active sites ascribed to the metal nodes (Zn^2+^ provides Lewis acid active site) and ligands (2-methylimidazole provides Bronsted basic active site) (Guan et al., 2005) of ZIF-8 (Zang *et al.* Zhang et al., 2011;^2017^; Qiu et al., 2021). The highly dispersed active centers of Au NPs are attributed to the inherent properties of ZIF-8 ordered porosity as well as uniform pore channels, and play an indispensable role in the effect of catalytic oxidation. Quite apart from that, the topographic hollow structure construction of catalysts displays surprising results in the catalytic performance of catalysts, which was crucial to improve the TOF values. By taking the structural advantages, the as-synthesized Void|(Au)ZIF-8|ZIF-8 catalyst exhibits an efficient multifunctional catalytic performance for rapid direct synthesis of imines from aniline and benzyl alcohol under mild conditions (air atmosphere, solvent-free and base-free).

## Experimental Sections

### Materials and Reagents

Sodium hydroxide (NaOH), ethanol (EtOH), Styrene (St), zinc nitrate hexahydrate (Zn(NO_3_)_2_·6H_2_O), hydrochloric acid (HCl, 37%), methanol (MeOH), Polystyrene pyrrolidone (PVP), Sulfuric acid (H_2_SO_4_), benzyl alcohol and N, N-Dimethylformamide (DMF) reagents were obtained from Sinopharm Chemical Reagent Co., Ltd. 2-methylimidazole (2-MI), Dodecane and amine were available from Beijing J&K Scientific, and sodium tetrachloroaurate (NaAuCl_4_∙2H_2_O) was obtained from Energy-Chemical.

### Synthesis and Purification of Sulfonated-Polystyrene Microspheres

Based on our group previous literature reported with slight modifications (Wan et al., 2017), in brief, 2.0 g polystyrene nanoparticles were mixed with 50 ml of concentrated H_2_SO_4_ ultrasound and then injected into a glass-made single-neck round bottom flask under 40°C water bath conditions, thereafter the reaction was maintained 24 h in a magnetic stirrer conditions. The final product was washed with deionized water and MeOH, and the white product was collected by centrifugation and eluted at 60°C vacuum drying oven for 12 h.

### Synthesis of SPS|ZIF-8 Nanospheres

In brief, 1.0 g SPS and 50 ml methanol were well dispersed by ultrasonication, and 100 ml methanol solution of 7.0 g 2-MI and 20.0 g PVP was injected into the reaction system at room temperature. After that, 50 ml of Zn(NO_3_)_2_·6H_2_O solutions was added dropwise and reacted for 24 h. The products were washed by centrifugation in MeOH, and subsequently dried with vacuum overnight at 80°C.

### Synthesis of SPS|(Au)ZIF-8

Typically, the activated SPS|ZIF-8 was dispersed in the EtOH solution of 20 ml of NaAuCl_4_ and treated for 30 s under ultrasonic conditions. After that, it was reacted at room temperature for 6 h under magnetic stirring condition. The impregnated samples were washed twice with ethanol, after which they were further dried at 60°C for 12 h, and the powder was reduced in a hydrogen reduction tube furnace at 250°C. Finally, incubation for 3 h yielded the SPS|(Au)ZIF-8.

### Synthesis of SPS|(Au)ZIF-8|ZIF-8 and Void|(Au)ZIF-8|ZIF-8.

In brief, 1.0 g SPS|(Au)ZIF-8 nanospheres were dispersed into 50 ml MeOH solution by ultrasonication, and 100 ml methanol solution of 7.0 g 2-MI and 20.0 g PVP was injected into the reaction system at room temperature. After that, 50 ml of Zn(NO_3_)_2_·6H_2_O solutions was added dropwise and reacted for 24 h. The products were washed by centrifugation in EtOH, after which they were further dried at 60°C overnight afforded SPS|(Au)ZIF-8|ZIF-8. The Void|(Au)ZIF-8|ZIF-8 nanospheres were synthesized by the way that SPS|(Au)ZIF-8|ZIF-8 was immersed in DMF to remove the polystyrene core template. Then, the final product was centrifuged. It was washed three times with methanol and vacuum dried at 60°C overnight for use.

### Synthesis of ZIF-8

Firstly, 5.0 g of 2-MI and 1.5 g of Zn(NO_3_)_2_·6H_2_O were dissolved separately in 50 ml of methanol solution. They were mixed well under mechanical stirring, and reacted for 3 h at room temperature to obtain ZIF-8 NPs. Then, the product was collected by centrifugation, washed several times with methanol, and dried overnight at 40°C.

### Synthesis of (Au)ZIF-8

Typically, the activated ZIF-8 NPs were mixed in 20 ml of a solution of NaAuCl_4_ under sonication conditions for 30 s. After that, the reaction was executed for 6 h at room temperature. The impregnated sample was washed with MeOH twice, followed by further drying at 60°C for 12 h, and the powder was reduced in a hydrogen reduction tube furnace at 250°C. Finally, incubation for 3 h yielded the (Au)ZIF-8.

### Catalytic Reactions

One-pot tandem/cascade catalysis reaction production of imines was carried out under a concentration condition (3.61 mmol of aniline/mg of Au). In the typical reaction system, a calculated amount of catalyst ((Au)ZIF-8, SPS|(Au)ZIF-8|ZIF-8 and Void|(Au)ZIF-8|ZIF-8) with the same Au content was dispersed in 2 ml benzyl alcohol. Then 0.625 mmol aniline and 0.25 mmol n-Dodecane as internal standard were injected into the above reaction system. Subsequently, the solution was transferred into an open round bottom flask and reacted under magnetic stirring in an air atmosphere at 60°C for 2 h. And 50 mg ZIF-8 was dispersed in 2 ml benzyl alcohol, and then 0.625 mmol aniline and 0.25 mmol n-Dodecane as an internal standard were injected into the above reaction system. Subsequently, the solution was transferred into an open round bottom flask and reacted under magnetic stirring in an air atmosphere at 60°C for 2 h. Subsequently, the recyclability tests were carried out. A calculated amount of Void|(Au)ZIF-8|ZIF-8 was added into the reaction system. The solution was transferred into an open round bottom flask and reacted under magnetic stirring in an air atmosphere at 60°C for 2 h. Finally, the solid catalyst was centrifugated and soaked with absolute ethanol to remove residual molecules of the reaction system. It was washed with absolute ethanol twice and dried at 40°C overnight under vacuum, after which it was reused under the uniform conditions.

### Material Characterization

The instruments were used for the analysis of properties such as morphology and structure: scanning electron microscopy (SEM, Zeiss sigma 300, and JSM-6701F), transmission electron microscopy (TEM, JEOL and JEM-2100F), X-ray diffraction (XRD, Rigaku SmartLab 9 kW diffractometer with Cu Kα radiation (*λ* = 1.541 Å), inductively coupled plasma (ICP, Agilent, ICP-OES-5100) emission spectroscopy, nitrogen adsorption-desorption measurement and multi-point BET (Brunauer-Emmett-Teller) (Quantachrome, Autosorb IQ/asiqwin analyzer), gas chromatography (Agilent GC-7820A) and gas chromatography-mass spectrometry (Agilent 7890B-5977MS).

## Results and Discussion

The synthesis procedures of the Void|(Au)ZIF-8|ZIF-8 catalysts were illustrated in Figure 1. With the assistance of hard template SPS, the inner ZIF-8 shell was perfectly constructed and named as SPS|ZIF-8. After that, Au NPs were cleverly confined to the ZIF-8 pore channels by a solution impregnation-reduction strategy to obtain SPS|(Au)ZIF-8. Sequentially, the outer ZIF-8 shell was grown stepwise on the surface of SPS|(Au)ZIF-8 to obtain SPS|(Au)ZIF-8|ZIF-8. Eventually, the hollow Void|(Au)ZIF-8|ZIF-8 was constructed by removing the hard template SPS (Wan et al., 2017; Zhong et al., 2019).

**FIGURE 2:**
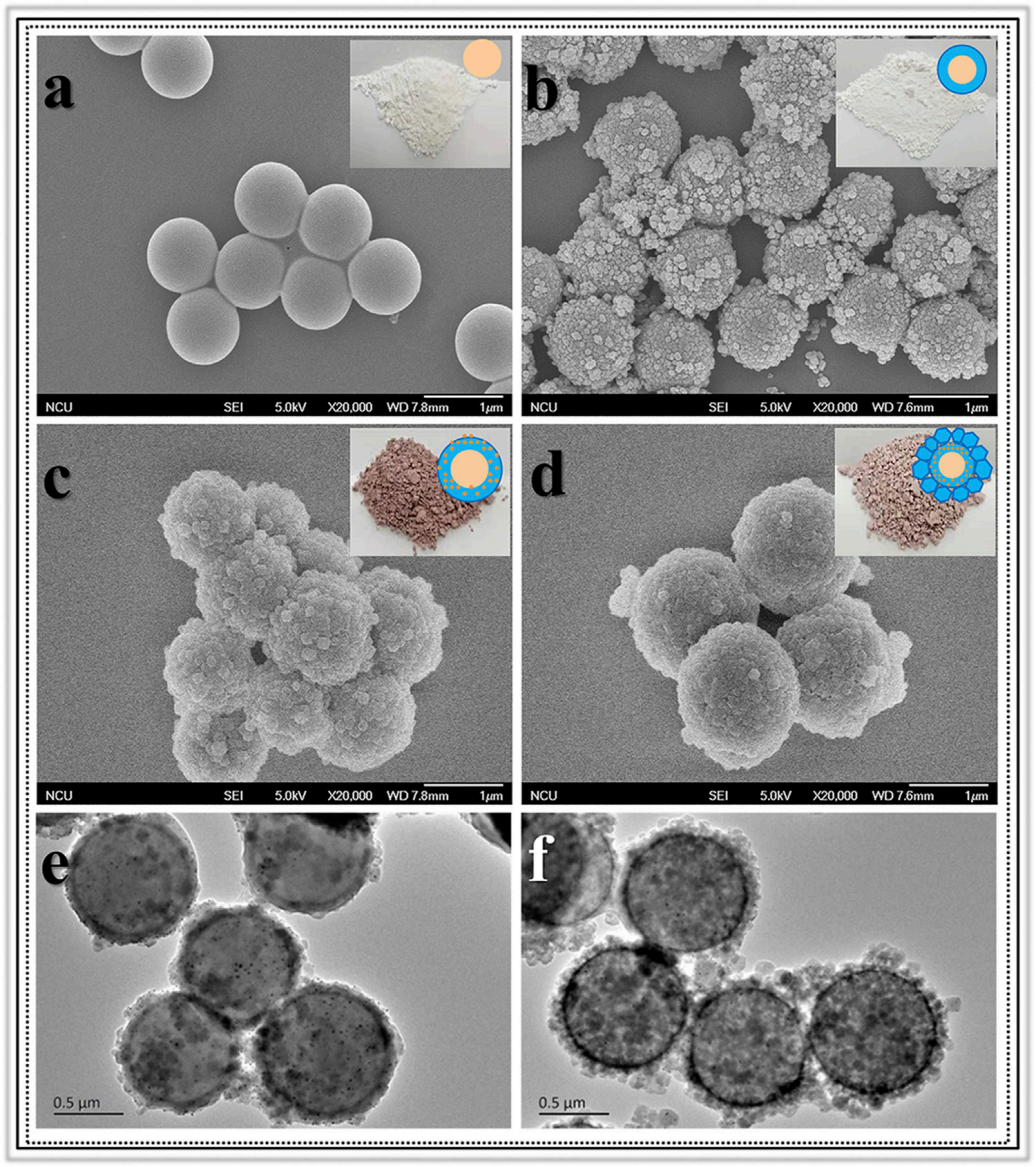
Images of SEM for **(A)** SPS; **(B)** SPS|ZIF-8; **(C)** SPS|(Au)ZIF-8; **(D)** SPS|(Au)ZIF-8|ZIF-8 and TEM for **(E)** SPS|(Au)ZIF-8; **(F)** SPS|(Au)ZIF-8|ZIF-8.

**FIGURE 3:**
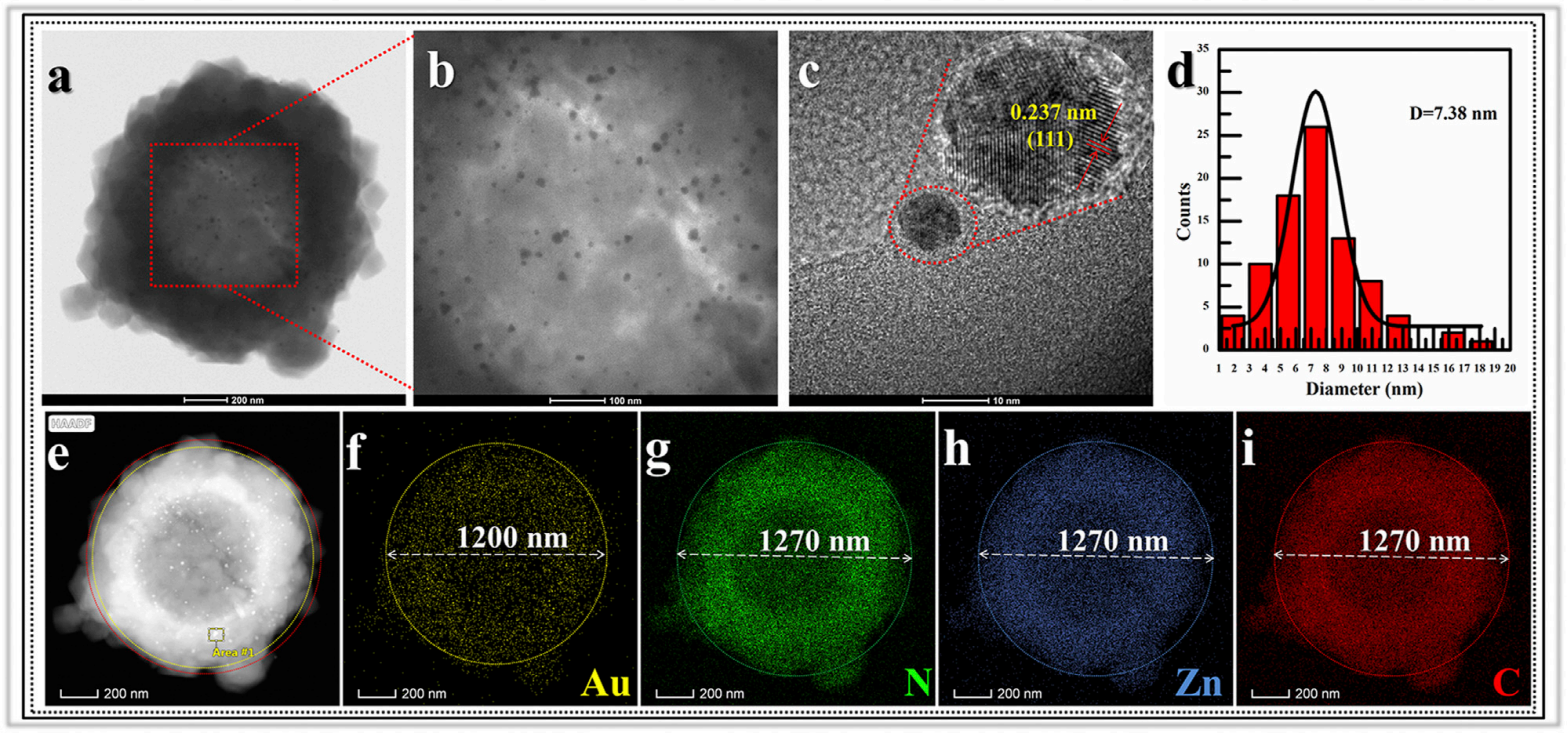
TEM images for **(A)** Void|(Au)ZIF-8|ZIF-8 and its **(B)** partial magnification image; **(C)** images of lattice fringes of Au nanoparticles and its **(D)** statistical image of the size distribution; **(E)** EDS-HAADF mapping images of **(F)** Au element; **(G)** N element; **(H)** Zn element and **(I)** C element.

**FIGURE 4:**
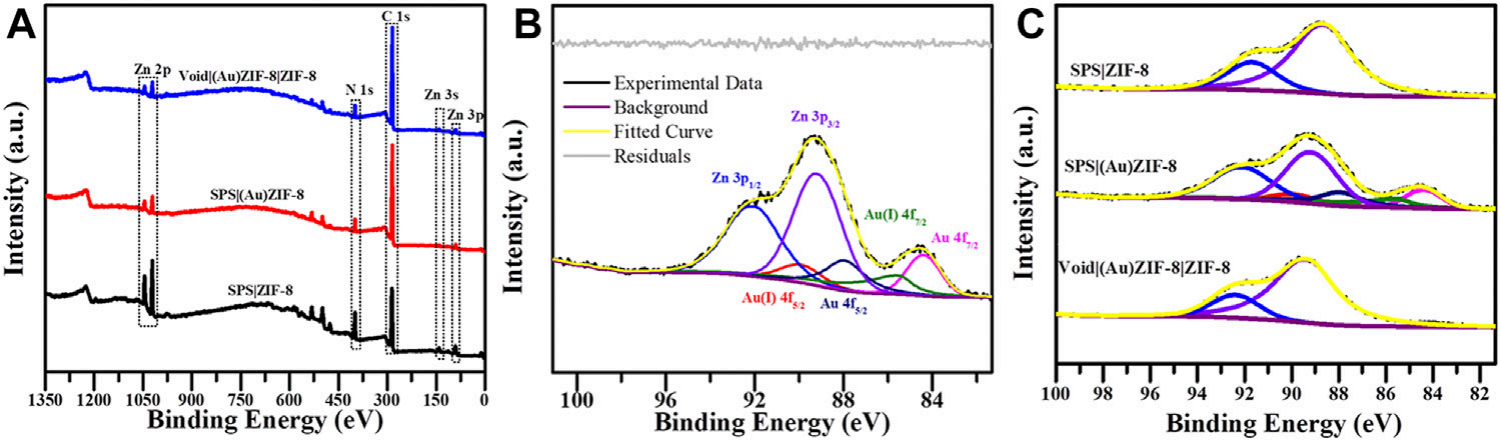
**(A)** XPS wide scan spectra of SPS|ZIF-8, SPS|(Au)ZIF-8 and Void|(Au)ZIF-8|ZIF-8. **(B)** High-resolution XPS spectrum for Au 4f and Zn 3p of SPS|(Au)ZIF-8. **(C)** High-resolution XPS spectrum of SPS|ZIF-8, SPS|(Au)ZIF-8 and Void|(Au)ZIF-8|ZIF-8.

Morphologic and structural analyses were monitored by SEM, TEM, XRD and Nitrogen adsorption-desorption characterizations. The SEM image information revealed the formation progress of Void|(Au)ZIF-8|ZIF-8. As shown in Figure 2, it demonstrated the successful growth of MOF shells on SPS surfaces (Figures 2A,B) via varying degrees of surface smoothness (Smooth SPS *vs* Rough SPS|ZIF-8). This result was attributed to the enrichment of metal cations by the -SO_3_H groups on the surface of SPS, which made the ligands easily undergo coordination polymerization with metal cations on the SPS surface. Further, TEM images (Figures 2E,F) showed the presence of shell structure on the surface of SPS, and the cavities (Figure 3A) were obviously found after removing the SPS template, implying the hollow structures of Void|(Au)ZIF-8|ZIF-8 were successfully constructed. Moreover, the Au NPs were obviously exhibited in the inner ZIF-8 shell (Figure 2C), while hardly be found in the outer shell (Figures 2F, 3A). The distribution ranges of C, N, and Zn were slightly wider than that of the Au element (diameter of ≈1,270 vs 1,200 nm), indicating that the outer shell was about 35 nm. As a further proof, the XPS (Figure 4) characterization was implemented on SPS|ZIF-8, SPS|(Au)ZIF-8 and Void|(Au)ZIF-8|ZIF-8 (Qian et al., 2008). Thus, it was demonstrated that Au NPs were confined in the first ZIF-8 shell, which implicitly indicated a double shell structure of Void|(Au)ZIF-8|ZIF-8. Furthermore, as a more intuitive way of expression, the macroscopic color change of images from white (SPS|ZIF-8) to light pink (SPS|(Au)ZIF-8), corroborating that the Au NPs have been encapsulated in the ZIF-8 shell. TEM image simultaneously revealed that the mean particle diameter of Au NPs was ca. 7 nm (Figures 3B,D) by statistical analysis. The 111) plane (Gumus et al., 2021) of Au existed in the HRTEM image (Figure 3C) of Void|(Au)ZIF-8|ZIF-8.

**FIGURE 5:**
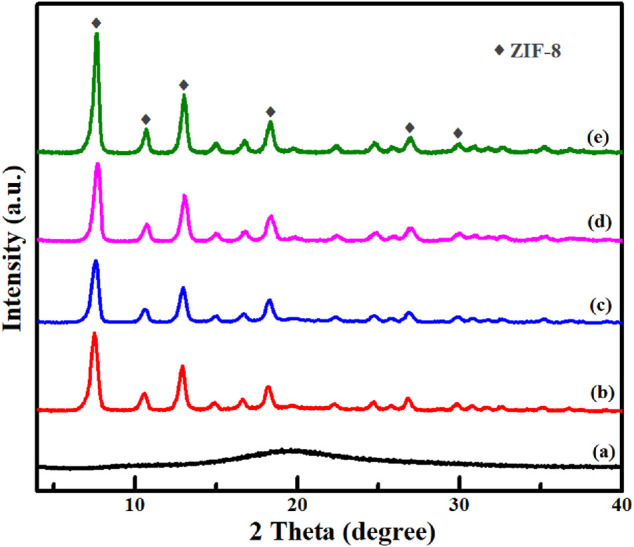
Powder XRD patterns of **(A)** SPS; **(B)** SPS|ZIF-8; **(C)** SPS|(Au)ZIF-8; **(D)** SPS|(Au)ZIF-8|ZIF-8 and **(E)** Void|(Au)ZIF-8|ZIF-8.

As shown in Figure 5, SPS had an amorphous structure from XRD pattern. However, it displayed obviously well-defined diffraction peaks of ZIF-8 after the MOF shell was grown on the SPS surface. Almost identical XRD patterns with ZIF-8 were obtained for SPS|ZIF-8, SPS|(Au)ZIF-8, SPS|(Au)ZIF-8|ZIF-8 and Void|(Au)ZIF-8|ZIF-8, indicating that the ZIF-8 shell was successfully constructed. Meanwhile, it was also implicitly illustrated a good maintenance of the crystallinity of Void|(Au)ZIF-8|ZIF-8 after removing the SPS template. No clear diffraction peaks of Au could be observed in the Void|(Au)ZIF-8|ZIF-8 sample, which suggested a low content of Au element (approximately 0.7% confirmed by ICP analysis, Supplementary Table S2) and small size of the Au NPs due to the lower detection resolution limit of the XRD technique.

**FIGURE 6:**
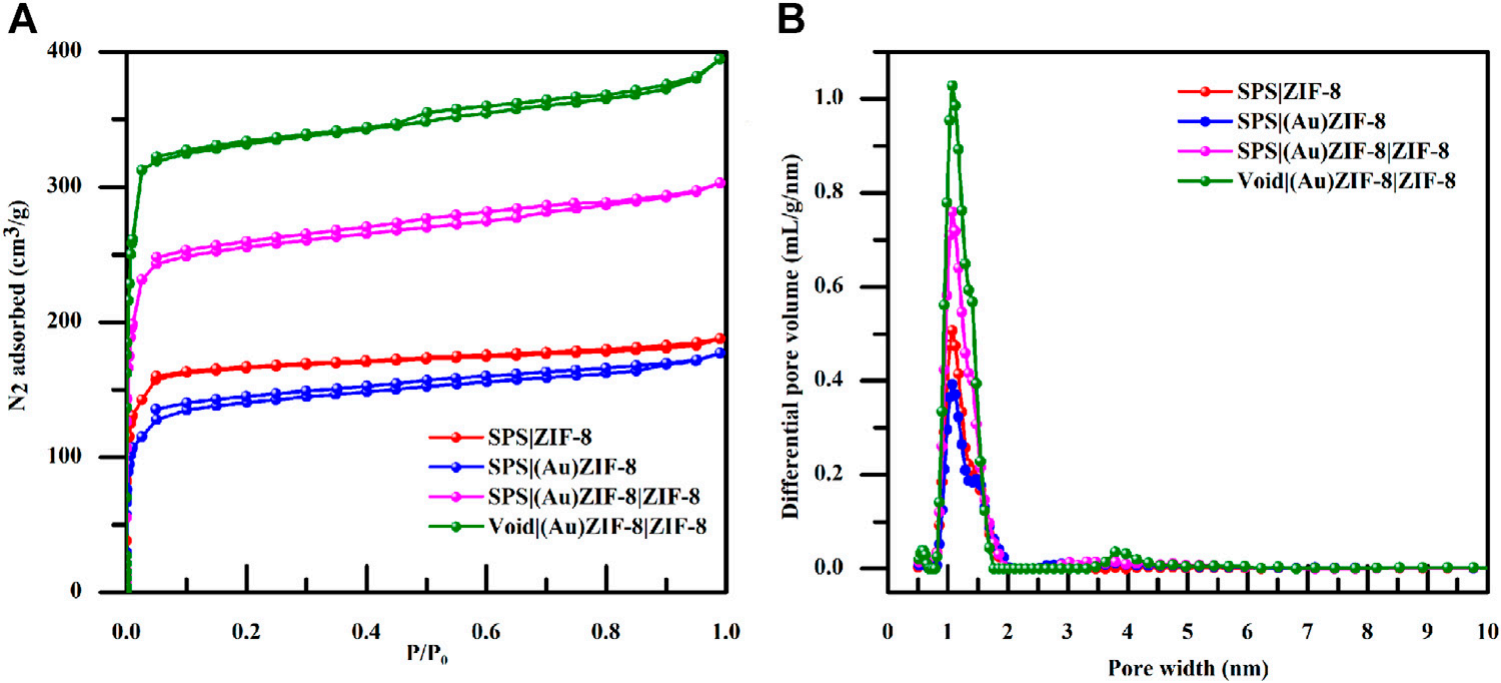
**(A)** Dinitrogen isotherms of SPS|ZIF-8 (red); SPS|(Au)ZIF-8 (blue); SPS|(Au)ZIF-8|ZIF-8 (magenta) and Void|(Au)ZIF-8|ZIF-8 (olive). **(B)** Pore size distribution image information of SPS|ZIF-8 (red); SPS|(Au)ZIF-8 (blue); SPS|(Au)ZIF-8|ZIF-8 (magenta) and Void|(Au)ZIF-8|ZIF-8 (olive).

To further explore the pore structure of the samples at each stage, nitrogen adsorption-desorption measurements were performed as an effective means of characterization. As shown in Figure 6A, both the surface area and pore volume of SPS|(Au)ZIF-8 decreased, which was ascribed to the occupation of pore channels by Au NPs after loading Au with initial sample SPS|ZIF-8. Moreover, as shown in Table 1, the surface area of SPS|ZIF-8 was reduced from 667 to 536 m^2^/g after loading Au NPs, and the corresponding pore volume was reduced by 0.017 cm^3^/g. However, as the synthesis progressed, the surface area of SPS|(Au)ZIF-8|ZIF-8 rose sharply after the growth of the outer ZIF-8 shell (from 536 m^2^/g of SPS|(Au)ZIF-8–1,041 m^2^/g of SPS|(Au)ZIF-8|ZIF-8). In addition, the corresponding pore volume increased to 0.410 cm^3^/g because of the high surface area and porosity of ZIF-8 shell. Inspiringly, the surface area and pore volume of Void|(Au)ZIF-8|ZIF-8 were further increased after template removal. Meanwhile, a significant hysteresis loop emerged from the nitrogen adsorption-desorption isotherm, further demonstrated that the product Void|(Au)ZIF-8|ZIF-8 possessed hollow structures. As shown in Figure 6B, the samples in the process of construction maintained micropore characteristics.

**TABLE 1:** The summary of surface area and pore volume for each step product.

Sample	S_BET_ ^a^ (m^2^/g)	V_pore_ ^b^ (cm^3^/g)
SPS|ZIF-8	667	0.256
SPS|(Au)ZIF-8	536	0.239
SPS|(Au)ZIF-8|ZIF-8	1,041	0.410
Void|(Au)ZIF-8|ZIF-8	1,388	0.528

a BET surface area calculated from the linear part of the BET plot.

b Single point total pore volume of pores at P/P_0_ = 0.99.

Then, as shown in Supplementary Figure S1, the pre-synthesized catalyst of Void|(Au)ZIF-8|ZIF-8 was applied to one-pot cascade catalytic imine synthesis reaction from aniline and benzyl alcohol under mild conditions (air atmosphere, solvent-free and base-free). As shown in Table 2, the catalytic cascade reaction with pure ZIF-8 was performed under an air atmosphere, solvent-free, 60°C for 2 h, which was subjected to GC and GC-MS analysis with almost no aniline conversion (Entry 1, Table 2). However, the catalyst of (Au)ZIF-8 (Entry 2, Table 2) gave 20% conversion of aniline and >99% selectivity under the same reaction conditions, which indicated that Au NPs played an important role in one-pot cascade catalysis. Then, one-pot cascade reaction catalyzed by SPS|(Au)ZIF-8|ZIF-8 (Entry 3, Table 2) was also experimented under the above reaction conditions. Despite the high selectivity, the conversion of aniline was 24%, which was comparable to that of the (Au)ZIF-8 (Con. 20% and Sel. >99%). This result was attributed to the presence of SPS which made SPS|(Au)ZIF-8|ZIF-8 similar to (Au)ZIF-8 without obvious hollow accelerated mass transfer effect (Zhong et al., 2019). Additionally, the slight dissolution of SPS in the reaction system would greatly hinder the delivery of the reaction substrate and reduce the contact frequency of the reaction substrate with the catalytic active center in unit time. In view of this, we embarked on building Void|(Au)ZIF-8|ZIF-8 by removing the inner core SPS. After that, the Void|(Au)ZIF-8|ZIF-8 was applied to catalyze the one-pot cascade reaction for imine synthesis (Entry 4, Table 2). Inspiringly, aniline was almost completely converted to imine under the above reaction conditions. The conversion was 99% and the product selectivity was more than 99% by GC and GC-MS analysis. Notably, it obtained a high TOF of 170.16 h^−1^, which was 2.15 times higher than that of the catalyst with the highest TOF value in the reported literatures (Cui et al., 2014). This result was attributed to the effect of hollow Void|(Au)ZIF-8|ZIF-8 accelerating the mass transfer, which greatly enhanced the collision odds between reactants and active sites in unit time, and rapidly oxidized benzyl alcohol to benzaldehyde under the catalysis of Au NPs. Additionally, there would not be any competitive adsorption of other solvent molecules with benzyl alcohol on the catalyst under solvent-free conditions, which would allow all active sites of catalytic to be fully exploited (Enache et al., 2006; Choudhary et al., 2007). After that, benzaldehyde underwent a condensation coupling reaction with aniline under the affection of ZIF-8 Lewis acidic sites.

**TABLE 2:** Synthesis of imines from benzyl alcohol and aniline by Void|(Au)ZIF-8|ZIF-8 one-pot cascade catalysis.

Entry	Cat	Time (h)	Con.(%)^b^	Sel.(%)^b^	TOF.(h^−1^)	Ref
1	ZIF-8^a^	2	trace	—	—	This work
2	(Au)ZIF-8	2	20	>99	79.32	This work
3	SPS|(Au)ZIF-8|ZIF-8	2	24	>99	40.79	This work
4	Void|(Au)ZIF-8|ZIF-8^a^	2	99	>99	170.16	This work
6	Au/MIL-101^c^	8	99	>99	51.47	Gumus *et al.* (2021)
7	Au–Pd@ZrO_2_ ^d^	7	91	97	79	Cui *et al.* (2014)
8	Au/Zn_0.02_Al_2_O_3_ ^e^	8	100	>99	39.1	Wu, Sun, et al. (2019)

a Void|(Au)ZIF-8|ZIF-8 (50 mg), benzyl alcohol (5 ml), amine (625 mmol), air. TOF is based on the ratio of the amounts of converted aniline to the amount of Au in the unit reaction time.

b Determined by performing GC analysis and confirmed by GC-MS using an internal standard.

c 3.0%-Au/MIL-101 (20 mg), amine (0.625 mmol), benzyl alcohol (0.625 mmol), *t-*BuOK (0.20 mmol), Toluene (5 ml), 343 K, Open flask/Ar.

d Benzyl alcohol (3 mmol), ethanol (10 ml); catalyst (50 mg), 40°C.

e Benzyl alcohol (0.5 mmol), aniline (0.75 mmol), catalyst (40 mg), toluene (5 ml), air (1 atm), 60°C.

**FIGURE 7:**
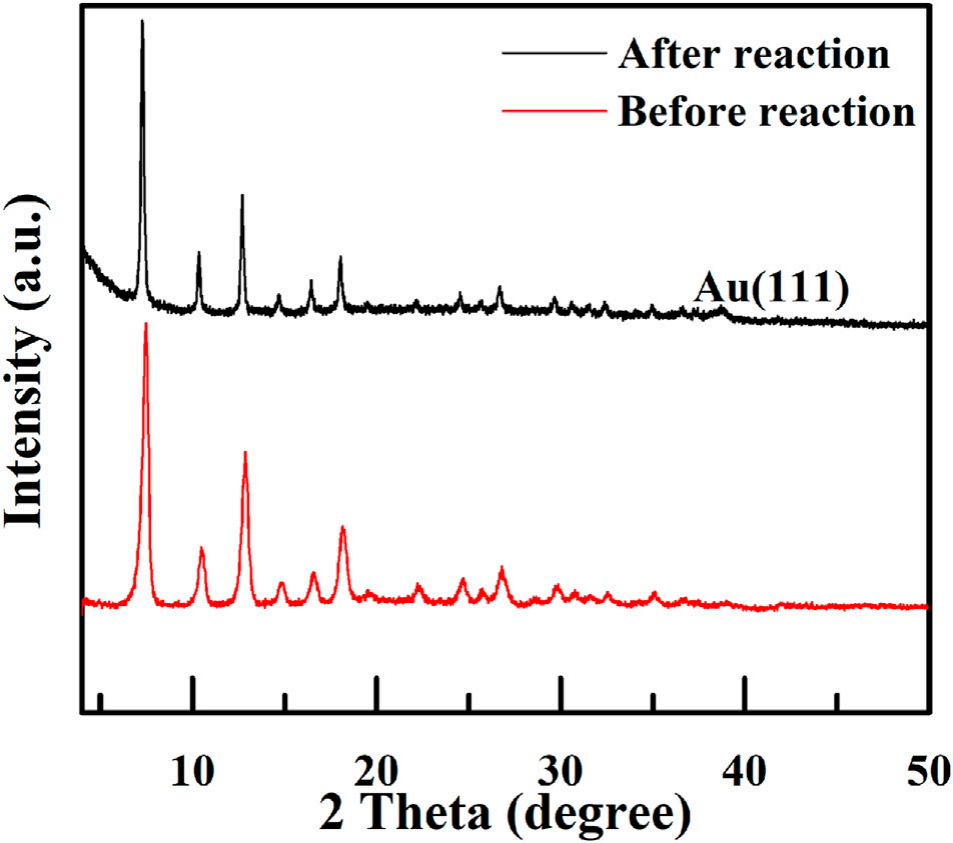
XRD patterns of Void|(Au)ZIF-8|ZIF-8 before and after reaction for the one-pot cascade catalytic synthesis of imines from benzyl alcohol and aniline.

We also performed cycling experiments for catalyst Void|(Au)ZIF-8|ZIF-8 under an air atmosphere, solvent-free and base-free, 60°C for 2 h, which unfortunately showed a decrease in performance upon a second cycle test. XRD (Figure 7) characterization of the samples before and after the reaction showed that the characteristic diffraction peaks of Au (111) appeared in the 2*θ* = 38.1° position (Yan et al., 2014), which indicated that the agglomeration of Au NPs was not conducive to the normal oxidation, resulting in the decrease of the turnover number in the unit time. We have carried out nitrogen adsorption-desorption experiments (Supplementary Figure S2) on the catalyst (Void|(Au)ZIF-8|ZIF-8 before and after reaction) to investigate the reduction of catalyst activity, which was conformed with the recession of micropore content and surface area with ca. 200 m^2^g. There were only trace amounts of Au element analyzed by ICP. Consequently, the slight deactivation may be deciphered with the blocking of the pore channels and the agglomerate of Au NPs leading to the decrease of the surface area and pore content. However, beyond delicate issues, we believe that constructing such novel structured catalyst Void|(Au)ZIF-8|ZIF-8 will be one of the most effective strategies to improve TOF values. Undoubtedly, this will inspire us to explore and construct catalysts with more delicate structures for better catalytic performance.

## Conclusion

In conclusion, we fabricated a delicately structured double MOFs shells catalyst of Void|(Au)ZIF-8|ZIF-8 via the hard template method. The pre-synthesized catalyst Void|(Au)ZIF-8|ZIF-8 could effectively promote the one-pot cascade catalytic synthesis of imines from benzyl alcohol and aniline under mild conditions. Our results confirmed that the hollow structure could accelerate the mass transfer, and the synergistic catalysis of Au nanoparticles with ZIF-8 was critical for the one-pot cascade reaction. We anticipate that the structure and synergistic effect of catalysts on improving the TOF of one-pot tandem/cascade reactions will be one of the future directions of development. Furthermore, multifunctional hollow double shell MNPs@MOF catalysts are expected to have broad application prospects in one-pot tandem/cascade reactions and even bionic catalysis for the synthesis of organic chemicals in the future.

## Data Availability

The original contributions presented in the study are included in the article/Supplementary Material, further inquiries can be directed to the corresponding authors.
