# Supplementary material for: Step by Step Construction of Multifunctional Hollow Double Shell MNPs@MOF as a Powerful Tandem/Cascade Catalyst
Source: Front Chem. 2021 Sep 15;9:738736. doi: 10.3389/fchem.2021.738736 (PMC8479107; doi:10.3389/fchem.2021.738736)
Supplement: Supplementary file 1 [file DataSheet1.docx]

Supplementary Material

**Step by Step Construction of Multifunctional Hollow Double Shell MNPs@MOF as a Powerful Tandem/Cascade Catalyst**

**Shunli Shi, Ying Yu, Bingzhen Zhang, Yicheng Zhong, Lei Wang, Shuhua Wang*, Shunmin Ding, Chao Chen***

*Key Laboratory of Jiangxi Province for Environment and Energy, College of Chemistry, Nanchang University, Nanchang, Jiangxi 330031, P. R. China.*

E-mail: chaochen@ncu.edu.cn; shwang@ncu.edu.cn

**Table S1.** Synthesis of imines from benzyl alcohol and aniline by heterogeneous Au/carriers catalysts one pot cascade catalysis

| **Entry** | **Cat.** | **Base** | **Solvent** | **[O]** | **Temp. (°C)** | **Time (h)** | **Con.(%)** | **Sel.(%)** | **TOF.(h^-1^)** | **Ref.** |
| --- | --- | --- | --- | --- | --- | --- | --- | --- | --- | --- |
| **1** | **Au/TiO_2_** | **KOCH_3_** | **MeOH** | **O_2_** | **RT** | **24** | **7** | **99** | **0.72** | **(Kegnæs *et al.* 2010)** |
| **2** | **Au–Pd@ZrO_2_** | **None** | **EtOH** | **air** | **40** | **7** | **91** | **97** | **79** | **(Cui *et al.* 2014)** |
| **3** | **Au/Zn_0.02_Al_2_O_3_** | **None** | **toluene** | **Air** | **60** | **8** | **99** | **>99** | **39.1** | **(Wu *et al.* 2019)** |
| **4** | **Au/HAP** | **None** | **toluene** | **O_2_** | **60** | **3** | **99** | **>99** | **16.34-** | **(Sun *et al.* 2009)** |
| **5** | **Au/C** | **None** | **toluene** | **O_2_** | **60** | **3** | **1** | **99** | **0.17** | **(Sun *et al.* 2009)** |
| **6** | **Au/TiO_2_** | **None** | **toluene** | **O_2_** | **60** | **3** | **36** | **88** | **5.28** | **(Sun *et al.* 2009)** |
| **7** | **Au/Fe_2_O_3_** | **None** | **toluene** | **O_2_** | **60** | **3** | **10** | **99** | **1.65** | **(Sun *et al.* 2009)** |
| **8** | **Au/CeO_2_** | **None** | **toluene** | **O_2_** | **60** | **3** | **53** | **74** | **6.54** | **(Sun *et al.* 2009)** |
| **9** | **Au/β-Ga_2_O_3_** | **None** | **toluene** | **O_2_** | **60** | **3** | **13** | **97** | **2.1** | **(Sun *et al.* 2009)** |
| **10** | **PICB-Au/Pd** | **NaOH** | **THF/TFE** | **O_2_** | **30** | **12** | **-^a^** | **-(70)^b^** | **23** | **(Soulé *et al.* 2013)** |
| **11** | **Cu-MOF/Au–Pd** | **None** | **toluene** | **air** | **110** | **12** | **-^a^** | **-(91)^b^** | ***^c^** | **(Zhong *et al.* 2020)** |
| **12** | **0.75%Pd–Au@Mn-MOF** | **KOH** | **toluene** | **O_2_** | **110** | **30** | **99** | **>99** | **4.4** | **(Chen *et al.* 2017)** |
| **13** | **Au/MIL-101** | **Bu*^t^*OK** | **toluene** | **Open flask/Ar** | **70** | **8** | **99** | **>99** | **51.47** | **(Gumus *et al.* 2021)** |
| **14** | **Void\|(Au)ZIF-8\|ZIF-8** | **None** | **None** | **Air** | **60** | **2** | **99** | **>99** | **170.16** | **This work** |

^a^ “-“referred to unknown in literature.

^b^ “(91) and (70)“ referred to the yield of imine.

^c^ “*“referred to unknown and cannot be calculated.

**Table S2.** The content of Au (ICP analysis) in catalysts (Au)ZIF-8, SPS|(Au)ZIF-8|ZIF-8 and Void|(Au)ZIF-8|ZIF-8.

| **Entry** | **Sample** | **Content (wt%)** |
| --- | --- | --- |
| 1 | (Au)ZIF-8 | 0.51 |
| 2 | SPS\|(Au)ZIF-8 | 0.90 |
| 2 | SPS\|(Au)ZIF-8\|ZIF-8 | 0.72 |
| 3 | Void\|(Au)ZIF-8\|ZIF-8 | 0.70 |

**Table S3.** The summary of surface area, pore volume and catalytic performance for each step product

| **Sample** | **S_BET_^a^ (m^2^/g)** | **V_pore_^b^ (cm^3^/g)** | **Con.(%)^c^** | **Sel.(%)^c^** | **TOF.(h^-1^)** |
| --- | --- | --- | --- | --- | --- |
| (Au)ZIF-8 | 1719 | 0.611 | 20 | >99 | 79.32 |
| SPS\|(Au)ZIF-8 | 536 | 0.239 | 17 | 98 | 45.58 |
| SPS\|(Au)ZIF-8\|ZIF-8 | 1041 | 0.410 | 24 | >99 | 40.79 |
| Void\|(Au)ZIF-8\|ZIF-8 | 1388 | 0.528 | 99 | >99 | 170.16 |

^a^ BET surface area calculated from the linear part of the BET plot.

^b^ Single point total pore volume of pores at P/P_0_ = 0.99.

^c^ Determined by performing GC analysis and confirmed by GC-MS using an internal standard. TOF is based on the ratio of the amounts of converted aniline to the amount of Au in the unit reaction time.

**Table S4.** The summary of Void|(Au)ZIF-8|ZIF-8 with different Au contents and their catalytic performance

| **Sample** | **Feeding Au^a^ (wt%)** | **Exact Au^b^ (wt%)** | **Con.(%)^c^** | **Sel.(%)^c^** | **TOF.(h^-1^)** |
| --- | --- | --- | --- | --- | --- |
| Void\|(Au)ZIF-8\|ZIF-8-0.37 | 0.5 | 0.37 | 82 | >99 | 140.94 |
| Void\|(Au)ZIF-8\|ZIF-8-0.7 | 1.0 | 0.70 | 99 | >99 | 170.16 |
| Void\|(Au)ZIF-8\|ZIF-8-1.32 | 1.5 | 1.32 | 79 | 96 | 130.35 |

^a^ The feeding Au loading for all samples.

^b^ The exact Au loading analyzed from ICP.

^c^ Determined by performing GC analysis and confirmed by GC-MS using an internal standard. TOF is based on the ratio of the amounts of converted aniline to the amount of Au in the unit reaction time.


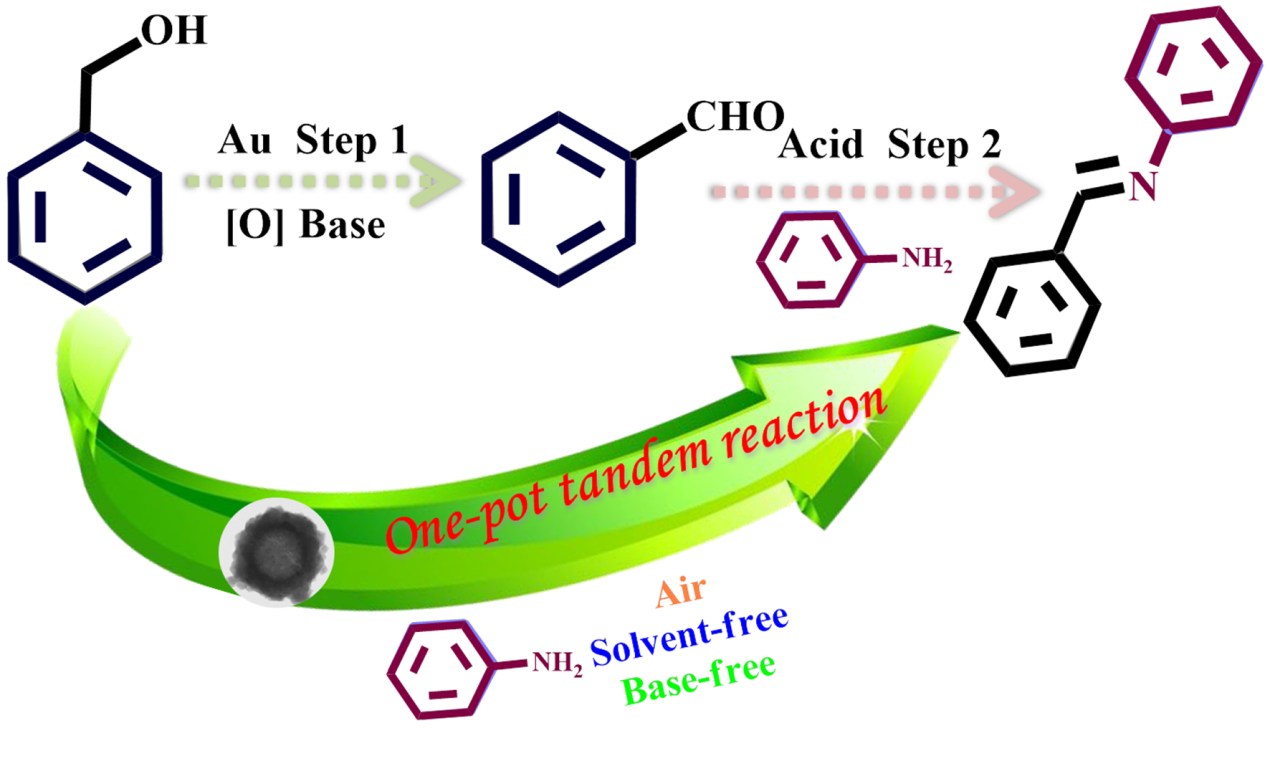


**Figure S1** Schematic application of Void|(Au)ZIF-8|ZIF-8 for one-pot cascade catalytic synthesis of imines from aniline and benzyl alcohol.





| Sample | S_BET_^a^ (m^2^/g) | V_pore_^b^ (cm^3^/g) |
| --- | --- | --- |
| Before reaction | 1388 | 0.528 |
| After reaction | 1194 | 0.498 |

**Figure S2** Dinitrogen isotherms and pore size distribution image information of Void|(Au)ZIF-8|ZIF-8 before and after reaction for the one-pot cascade catalytic synthesis of imines from benzyl alcohol and aniline.

**References**

Kegnæs, S., Mielby, J., Mentzel, U.V., Christensen, C.H., and Riisager, A. (2010). Formation of imines by selective gold-catalysed aerobic oxidative coupling of alcohols and amines under ambient conditions. *Green Chem.* 12 (8). 1437-1441. doi: 10.1039/C0GC00126K.

Cui, W., Xiao, Q., Sarina, S., Ao, W., Xie, M., Zhu, H., and Bao, Z. (2014). Au–Pd alloy nanoparticle catalyzed selective oxidation of benzyl alcohol and tandem synthesis of imines at ambient conditions. *Catal. Today* 235. 152-159. doi: 10.1016/j.cattod.2014.04.015.

Wu, S., Sun, W., Chen, J., Zhao, J., Cao, Q., Fang, W., and Zhao, Q. (2019). Efficient imine synthesis from oxidative coupling of alcohols and amines under air atmosphere catalysed by Zn-doped Al_2_O_3_ supported Au nanoparticles. *J. Catal.* 377. 110-121. doi: 10.1016/j.jcat.2019.07.027.

Sun, H., Su, F.-Z., Ni, J., Cao, Y., He, H.-Y., and Fan, K.-N. (2009). Gold supported on hydroxyapatite as a versatile multifunctional catalyst for the direct tandem synthesis of imines and oximes. *Angew. Chem. Int. Ed.* 48 (24). 4390-4393. doi: 10.1002/anie.200900802.

Soulé, J.-F., Miyamura, H., and Kobayashi, S. (2013). Selective imine formation from alcohols and amines catalyzed by polymer incarcerated gold/palladium alloy nanoparticles with molecular oxygen as an oxidant. *Chem. Commun.* 49 (4). 355-357. doi: 10.1039/C2CC36213A.

Zhong, M., Zhang, S., Dong, A., Sui, Z., Feng, L., and Chen, Q. (2020). Cu-MOF/Au–Pd composite catalyst: preparation and catalytic performance evaluation. *Journal of Materials Science* 55 (24). 10388-10398. doi: 10.1007/s10853-020-04699-z.

Chen, G.-J., Ma, H.-C., Xin, W.-L., Li, X.-B., Jin, F.-Z., Wang, J.-S., Liu, M.-Y., and Dong, Y.-B. (2017). Dual heterogeneous catalyst Pd–Au@Mn(II)-MOF for one-pot tandem synthesis of imines from alcohols and amines. *Inorg. Chem.* 56 (1). 654-660. doi: 10.1021/acs.inorgchem.6b02592.

Gumus, I., Ruzgar, A., Karatas, Y., and Gulcan, M. (2021). Highly efficient and selective one-pot tandem imine synthesis via amine-alcohol cross-coupling reaction catalysed by chromium-based MIL-101 supported Au nanoparticles. *Mol. Catal.* 501 (5). 111363. doi: 10.1016/j.mcat.2020.111363.
